# NEURL1-mediated K63-ubiquitination of SLC40A1 drives ferroptosis resistance in pathological scars: Therapeutic targeting via autophagy-activating microneedles

**DOI:** 10.1016/j.redox.2026.104420

**Published:** 2026-09-29

**Authors:** Mengzhu Lv, Jiamei Liu, Qi Dong, Zibo Guo, Qianlong Wu, Simeng Zhang, Shengye Liu

**Affiliations:** a Department of Plastic Surgery, The First Hospital of China Medical University, Shenyang, Liaoning, 110001, China; b Department of Pathology, Shengjing Hospital of China Medical University, Shenyang, Liaoning, 110004, China; c Department of Orthopedics, Shengjing Hospital of China Medical University, Shenyang, Liaoning, 110004, China; d Department of Medical Oncology, The First Hospital of China Medical University, Shenyang, Liaoning, 110001, China

**Keywords:** Pathological scars, Ferroptosis, SLC40A1, Autophagy, Microneedles

## Abstract

Ferroptosis resistance contributes to pathological skin scarring, but the underlying regulatory mechanisms remain unclear. Here, we identified a novel selective autophagy pathway NEURL1-SQSTM1/p62-SLC40A1 that controls fibroblast ferroptosis and scar formation. SLC40A1 is upregulated in human scars, conferring ferroptosis resistance by promoting iron efflux. Mechanistically, NEURL1 mediates K63-linked ubiquitination of SLC40A1, enabling its recognition by SQSTM1 for lysosomal degradation. A microneedle system co-delivering curcumin (autophagy activator) and siSLC40A1 effectively activates this axis, induces ferroptosis, and alleviates scarring in vivo. Our findings reveal a new therapeutic target and strategy for pathological scars.

## Highlights

• SLC40A1 confers ferroptosis resistance to maintain skin scar fibrosis.
• A novel NEURL1-SQSTM1-SLC40A1 axis modulates scar ferroptosis and fibrosis.
• NEURL1 mediates SLC40A1 K63 ubiquitination and autophagic degradation.
• Curcumin inhibits scar fibrosis via NEURL1-mediated SLC40A1 degradation.

## Graphical abstract

**Left Panel (Pathological State):** In the hypertrophic scar microenvironment, myofibroblasts upregulate SLC40A1 expression. This increases iron efflux and concurrently inhibits autophagy, enabling the cells to evade ferroptosis. Consequently, this leads to persistent cell survival, excessive collagen secretion, and ultimately drives scar progression. **Right Panel (Therapeutic Outcome):** The microneedle-based combination therapy delivers curcumin and siSLC40A1. This strategy degrades SLC40A1, resulting in intracellular iron accumulation, and activates autophagy—a process critically involving SQSTM1 and NEURL1. These actions synergistically induce robust ferroptosis in myofibroblasts, thereby effectively inhibiting scar hyperplasia.

**Figure:**
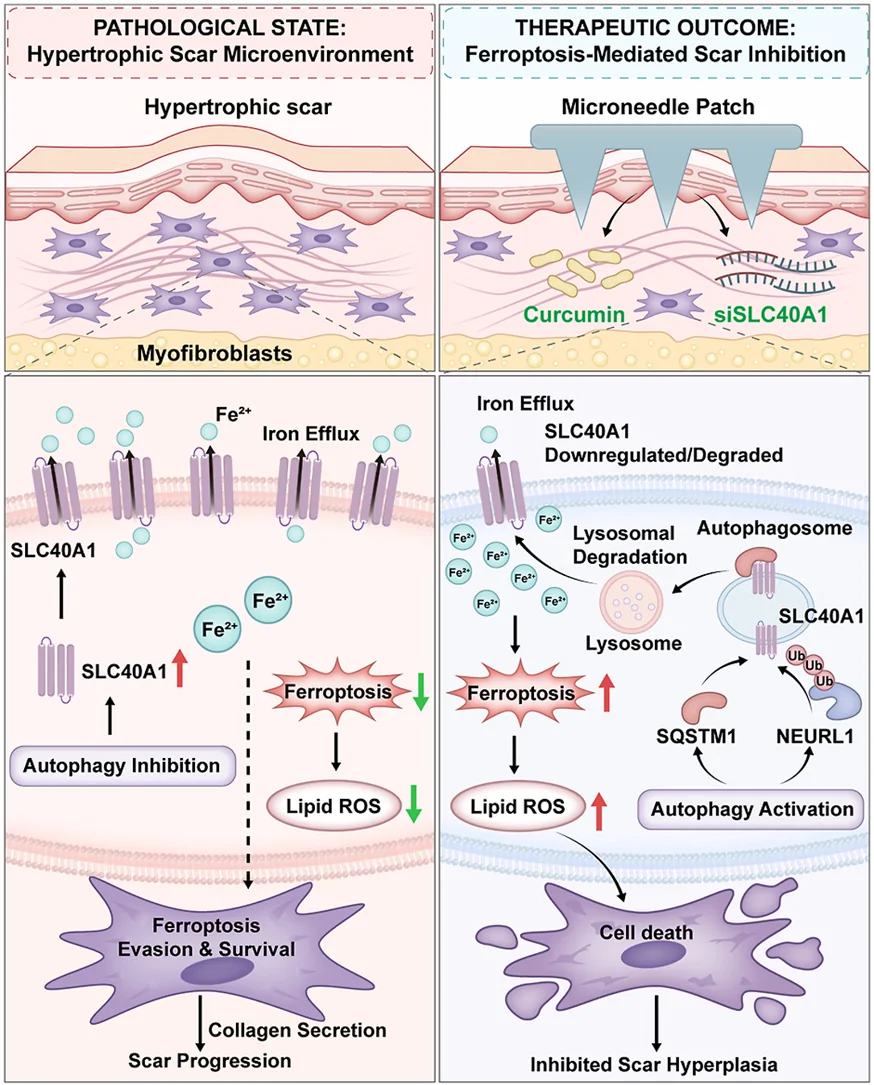

## 1 Introduction

The therapeutic challenge of hypertrophic scars and keloids stems from an insufficient understanding of the mechanisms behind the abnormal survival and persistent activation of dermal fibroblasts, the core effector cells [[1], [2], [3], [4], [5], [6]]. Emerging evidence indicates that ferroptosis, an iron-dependent form of cell death driven by lipid peroxidation, plays a crucial inhibitory role in fibrosis of organs such as the liver and lungs, suggesting its potential importance in skin fibrosis [[7], [8], [9], [10], [11]]. Hence, a key scientific question is whether fibroblasts are capable of undergoing ferroptosis and whether this process is specifically suppressed in pathological scars. The iron exporter SLC40A1 (Ferroportin) is a central regulator of cellular iron homeostasis, and its dysregulation may directly determine cellular ferroptosis sensitivity [[12], [13], [14], [15]]. Recent reviews have highlighted the critical role of ferroptosis in fibrotic diseases, but the regulatory networks in skin scarring remain understudied [16,17]. Therefore, investigating the expression, function, and upstream regulation of SLC40A1 in scar fibroblasts is of significant theoretical value and clinical urgency, as it may reveal whether scars persist by acquiring “ferroptosis resistance” and identify novel therapeutic targets.

While the involvement of ferroptosis in fibrosis is increasingly acknowledged, its specific role in skin scarring remains an area with limited investigation and elusive mechanistic understanding. Existing research in this context has been largely observational, often correlating scarring with general markers of oxidative stress, while deeper mechanistic insights into the execution or resistance pathways of ferroptosis are inadequate [[18], [19], [20], [21], [22]]. Separately, autophagy, a crucial intracellular regulatory and degradation system, has been implicated in iron metabolism through the processing of proteins like ferritin [[23], [24], [25]]. However, its potential to directly govern fibroblast ferroptosis by targeting the iron exporter SLC40A1 for degradation has not been investigated. Consequently, several key questions persist, including the identity of a specific molecular link between autophagy and fibroblast ferroptosis, the precise mechanism regulating SLC40A1 by autophagy in scars, and the absence of therapeutic strategies designed to target this regulatory axis.

To address these gaps, we proposed a hypothesis: the long-term survival of fibroblasts in pathological scars may stem from their acquired resistance to ferroptosis, and this phenotype is likely regulated by the stability of specific iron metabolism proteins. The iron exporter SLC40A1 (Ferroportin) is a key player in maintaining cellular iron homeostasis [12]. Its abnormal accumulation might lead to “functional iron depletion,” thereby inhibiting ferroptosis. Therefore, elucidating the regulatory mechanism and function of SLC40A1 in scar fibroblasts represents a potential key to understanding and intervening in the fibrotic process. This study aimed to systematically validate the role of SLC40A1 in scarring and, for the first time, investigate whether its expression is meticulously regulated by the autophagy-ubiquitination system, thereby bridging the two critical biological processes of autophagy and ferroptosis.

Integrating bioinformatics, clinical samples, cell models, and animal experiments, our study yielded several key findings: 1) SLC40A1 is upregulated in pathological scars and associated with ferroptosis resistance; 2) Functionally, SLC40A1 regulates ferroptosis sensitivity and activation in fibroblasts; 3) Mechanistically, the E3 ligase NEURL1 mediates K63-linked ubiquitination of SLC40A1, promoting its recognition and degradation by the autophagy receptor SQSTM1/p62 (sequestosome 1), forming a novel regulatory axis; 4) Targeting this axis (e.g., with curcumin or a co-delivery system) effectively alleviated scar formation. This work unveils a new mechanism regulating ferroptosis via autophagic degradation of SLC40A1, offering a novel therapeutic target for scarring.

## 2 Results

### 2.1 SLC40A1 mediates ferroptosis resistance in scar fibroblasts

To investigate the molecular mechanisms underlying pathological scarring, we performed an integrated analysis of public datasets. Differential gene expression analysis of datasets GSE72901 (low glucose-induced autophagy activation) and GSE178411 (late-vs. early-stage scars) (Fig. S1A and B) was conducted. Intersecting the results with a gene set related to ferroptosis inhibition identified the iron exporter SLC40A1 as a key candidate gene (Fig. S1C). Functional enrichment analysis suggested that the differentially expressed genes were associated with iron metabolism and oxidative stress pathways (Fig. S1D and E). Notably, SLC40A1 expression was upregulated in late-stage scar samples (characterized by autophagy suppression) but downregulated under autophagy-activating conditions (Fig. S1F), implying that its expression is regulated by both scar progression stage and autophagic status.

Building on these findings, a bioinformatics prediction model further supported the hypothesis that scar progression is accompanied by increased ferroptosis resistance (Fig. S1G). To validate this, clinical samples were collected for analysis, including 10 hypertrophic scar (HS), 10 keloid scar (KS) specimens, and paired normal skin tissues. Experimental validation confirmed a significant increase in SLC40A1 mRNA levels in scar-derived fibroblasts compared to normal tissues (Fig. S1H). This was accompanied by elevated iron deposition (Fig. S1I) and increased total iron content (Fig. S1J), yet a decrease in the level of the lipid peroxidation product malondialdehyde (MDA) (Fig. S1K), collectively indicating suppression of ferroptosis. Western blot analysis further elucidated the molecular basis: in scar tissues, the iron storage protein FTH1, and SLC40A1 were all upregulated (Fig. S1L). Concurrently, key negative regulators of ferroptosis, GPX4 and SLC7A11, were also significantly increased (Fig. S1L). These results suggest that in both hypertrophic scars and keloids, upregulation of SLC40A1 in fibroblasts remodels intracellular iron homeostasis, thereby conferring resistance to ferroptosis.

### 2.2 Curcumin attenuates pathological scarring by activating autophagy to downregulate SLC40A1 and induce ferroptosis

Building on the finding in Result 1 that SLC40A1 contributes to ferroptosis resistance, we next explored whether this resistance in fibroblasts could be reversed by promoting the autophagic degradation of SLC40A1. To this end, we selected curcumin (Cur), a known autophagy activator, and the classical ferroptosis inducer RSL3 for subsequent intervention studies [26,27]. To explore novel strategies for targeted intervention in scar formation, we compared the therapeutic effects of the autophagy activator Cur with the classical ferroptosis inducer RSL3 in a mouse model of hypertrophic scarring (Fig. 1A). Both macroscopic and histological evaluations confirmed that both treatments significantly improved scar appearance, reduced tissue thickness, and ameliorated collagen disorganization (Fig. 1B and C).

**Fig. 1:**
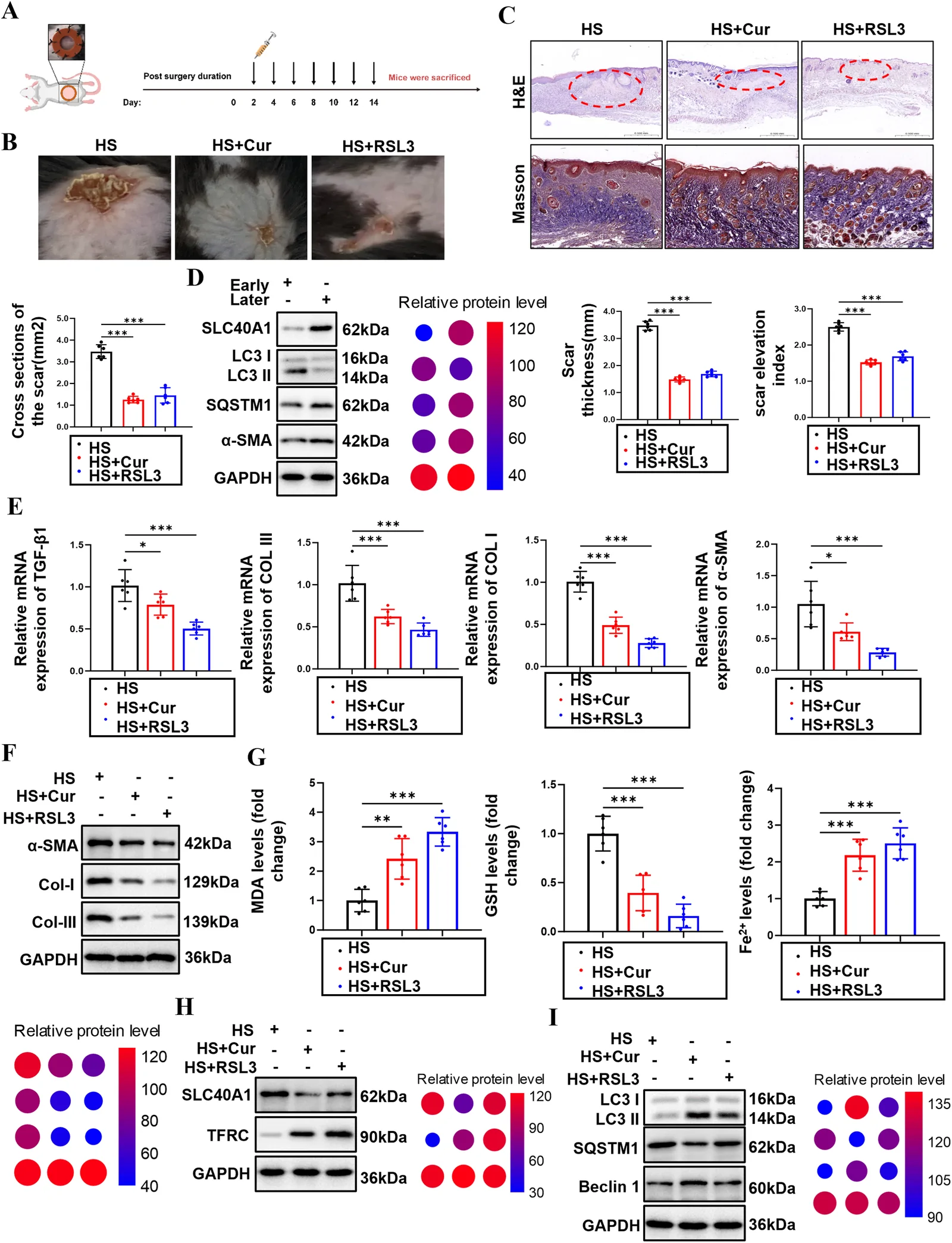
Curcumin and a ferroptosis inducer alleviate hypertrophic scars in mice by activating autophagy, downregulating SLC40A1, and promoting ferroptosis. (A) Schematic diagram of the mouse hypertrophic scar model and the treatment regimen with curcumin (Cur) or RSL3. (B) Representative macroscopic photographs of healed wounds from each group, showing scar appearance. (C) Histological analysis of wound tissue sections by Hematoxylin and Eosin (H&E) and Masson's trichrome staining. (D) Western blot results and corresponding heatmap showing the expression of autophagy-related (SQSTM1, LC3Ⅱ/Ⅰ) and fibrosis-associated (SLC40A1, α-SMA) proteins. (E) Quantitative analysis of relative mRNA expression levels of fibrosis-related genes (Tgfb1, Col3a1, Col1a1, Acta2). (F) Western blot analysis of myofibroblast activation and extracellular matrix proteins (α-SMA, Col-I, Col-III). (G) Biochemical assessment of ferroptosis-related indicators in tissues, including malondialdehyde (MDA), glutathione (GSH), and ferrous iron (Fe^2+^) levels. (H) Western blot analysis of iron metabolism-related proteins (SLC40A1, TFRC). (I) Western blot analysis of autophagic flux markers (LC3Ⅱ/Ⅰ, SQSTM1). Data are presented as mean ± SD (n = 6 mice per group). For histological quantification in (C), at least 5 randomly selected non-overlapping fields per section were analyzed using ImageJ software. Differences among multiple groups were analyzed by one-way ANOVA followed by Tukey's multiple comparison test (*p < 0.05, **p < 0.01, ***p < 0.001).

We further investigated the underlying molecular mechanisms. Western blot analysis revealed that both Cur and RSL3 effectively activated autophagic flux, as evidenced by increased LC3Ⅱ/Ⅰ and decreased SQSTM1, concurrently with the downregulation of the fibrosis-associated protein SLC40A1 (Fig. 1D–I). Accompanying this process, the expression of the myofibroblast activation marker α-SMA and extracellular matrix proteins (Col-I, Col-III) was significantly suppressed (Fig. 1D–F). Correspondingly, the transcriptional levels of fibrosis-related genes were markedly reduced (Fig. 1E).

Critically, both Cur and RSL3 treatments effectively promoted ferroptosis (Fig. 1G and H). Immunofluorescence staining further confirmed the decreased expression of the SLC40A1 protein in situ within the tissue (Fig. S2A). These results indicate that Cur can steer cellular fate towards ferroptosis by activating autophagy and subsequently downregulating SLC40A1. Furthermore, both treatments inhibited aberrant angiogenesis in the scars, demonstrated by reduced microvessel density (CD31-positive signals) and decreased VEGF levels (Fig. S2B and C). Collectively, these findings suggest that Cur exerts its anti-scarring effects by activating autophagy, downregulating SLC40A1 to induce ferroptosis, while simultaneously suppressing fibrosis and angiogenesis.

### 2.3 Knockdown of SLC40A1 attenuates fibroblast-to-myofibroblast transition and fibrotic progression by inducing ferroptosis

To investigate the direct role of SLC40A1 in fibroblast activation, studies were conducted using a TGF-β1-induced in vitro fibrosis model. Initially, immunofluorescence staining revealed a significant upregulation of SLC40A1 protein in activated scar-derived fibroblasts (HSFs), which exhibited pronounced colocalization with the myofibroblast marker α-SMA (Fig. 2A). Western blot analysis further confirmed that SLC40A1 expression accumulated in a dose- and time-dependent manner upon TGF-β1 stimulation (Fig. 2B), paralleled by an enhanced degree of colocalization with α-SMA (Fig. 2C), suggesting a close association between SLC40A1 and the fibroblast activation process.

**Fig. 2:**
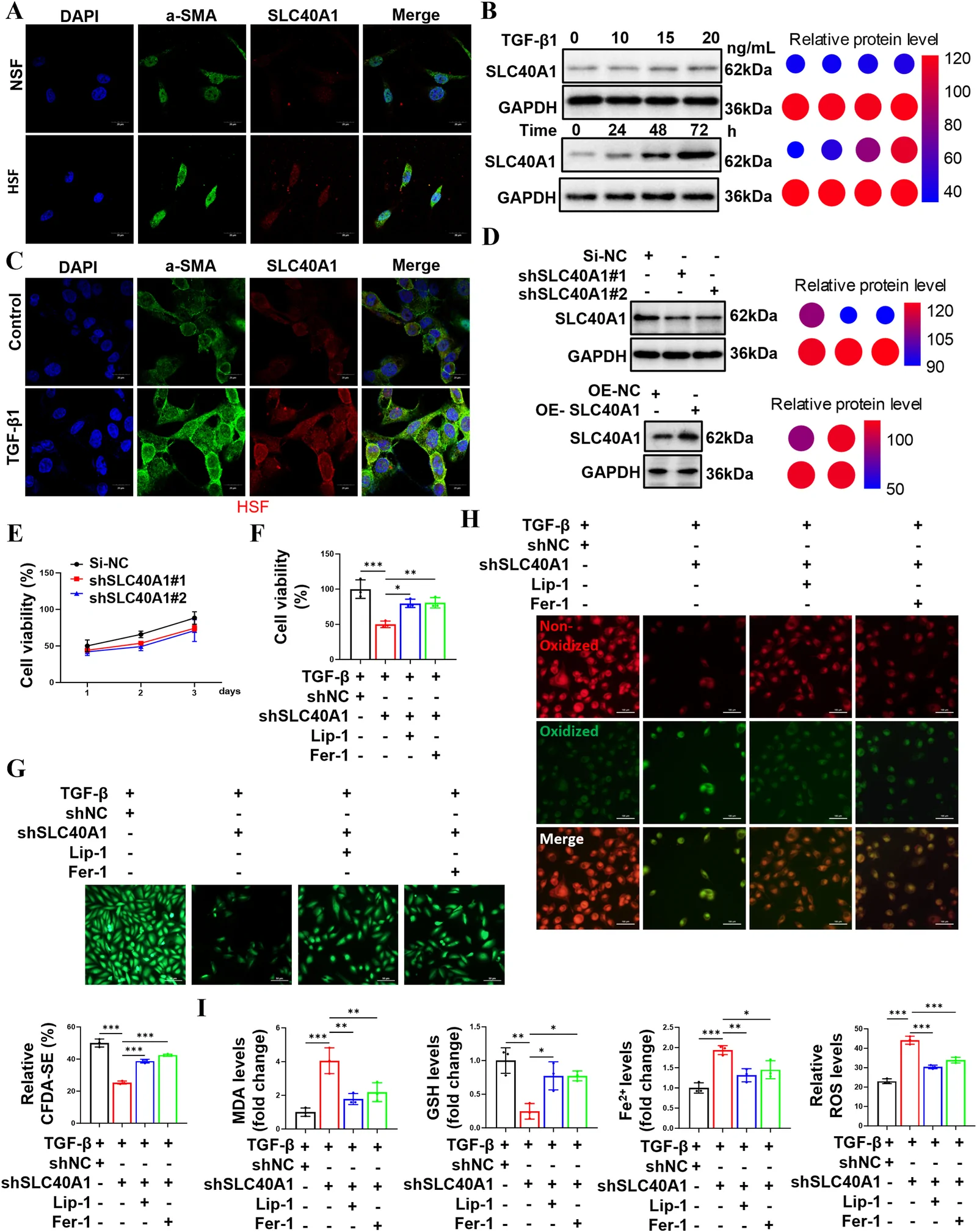
Knockdown of SLC40A1 inhibits TGF-β1-driven fibroblast activation by inducing ferroptosis. (A) Representative immunofluorescence images showing colocalization of α-SMA (green) and SLC40A1 (red) in normal skin fibroblasts (NSFs) and hypertrophic scar-derived fibroblasts (HSFs). (B) Western blot analysis of SLC40A1 protein expression in HSFs stimulated with TGF-β1 at different concentrations or for varying durations. (C) Quantitative analysis of the colocalization between SLC40A1 (red) and α-SMA (green) in HSFs following TGF-β1 treatment. (D) Western blot validation of SLC40A1 knockdown or overexpression in HSFs. (E) Effect of SLC40A1 knockdown on HSF viability (growth curve). (F) Bar graph comparing cell viability among the different experimental groups. (G) Proportion of viable cells as detected by CFDA-SE staining. (H) Intracellular oxidative stress levels visualized using a redox-sensitive fluorescent probe. (I) Quantitative results of key ferroptosis-related biochemical indicators (MDA, GSH, Fe^2+^, ROS). Data are expressed as mean ± SD from n = 3 independent biological experiments. For immunofluorescence colocalization and fluorescent probe analysis in (A, C, H), 5 random visual fields per sample were analyzed using ImageJ. Statistical significance was determined using two-tailed Student's t-test or one-way ANOVA with Tukey's post hoc test (*p < 0.05, **p < 0.01, ***p < 0.001).

To explore its functional role, a stable SLC40A1-knockdown (shSLC40A1) cell model was successfully established (Fig. 2D). Functional assays demonstrated that SLC40A1 knockdown significantly inhibited the TGF-β1-induced increases in cell viability (Fig. 2E and F), proliferation (CFDA-SE staining, Fig. 2G), and migratory capacity (wound healing and Transwell assays, Fig. S3A and B). More importantly, SLC40A1 knockdown effectively blocked the fibroblast-to-myofibroblast transdifferentiation, as evidenced by downregulated expression of key fibrotic proteins (α-SMA, COL-I, COL-III) and matrix metalloproteinases (MMP2/MMP9) (Fig. S3C–E).

The underlying mechanism was further explored. Knockdown of SLC40A1 effectively promoted ferroptosis (Fig. 2H and I). Notably, all phenotypes induced by SLC40A1 knockdown—including reduced cell viability (Fig. 2F), increased oxidative stress (Fig. 2H), inhibited migration (Fig. S3A and B), and decreased expression of fibrotic markers (Fig. S3C–E)—were significantly reversed by the specific ferroptosis inhibitors Liproxstatin-1 (Lip-1) or Ferrostatin-1 (Fer-1). These results indicate that SLC40A1 knockdown restrains TGF-β1-driven fibroblast activation and fibrotic progression primarily by triggering the ferroptotic cell death pathway.

### 2.4 Autophagy activation attenuates the pro-fibrotic and anti-ferroptotic functions of SLC40A1 via its degradation

To validate the function of SLC40A1 and explore potential intervention strategies, we performed overexpression studies in fibroblasts. In contrast to the knockdown results, overexpression of SLC40A1 in the context of TGF-β1 stimulation exacerbated fibrotic phenotypes, as demonstrated by enhanced cell migration (Transwell and scratch assays, Fig. S4F and H) and increased expression of the myofibroblast transdifferentiation marker α-SMA (Fig. S4G). Concurrently, SLC40A1 overexpression significantly inhibited ferroptosis (Fig. S4E), a phenotype consistent with observations in scar tissues in vivo, indicating that elevated SLC40A1 expression is pivotal for driving fibrosis and conferring ferroptosis resistance.

Notably, treatment with the autophagy activator rapamycin (Rap) completely reversed the spectrum of phenotypes induced by SLC40A1 overexpression. This reversal included the restoration of cell viability (Fig. S4A and B), alleviation of oxidative stress (Fig. S4C), suppression of cell migration and activation (Fig. S4F–H), and the re-establishment of ferroptosis susceptibility (Fig. S4E). Mechanistically, we found that rapamycin significantly reduced SLC40A1 protein levels (Fig. S4D), suggesting that autophagy activation may function, at least in part, by promoting SLC40A1 degradation. Furthermore, autophagy activation exhibited potent anti-fibrotic effects, as rapamycin treatment robustly and comprehensively suppressed the expression of key fibrotic proteins (Fig. S5A and B).

To substantiate this regulatory axis, we further assessed autophagic flux. Rapamycin effectively activated autophagy (Fig. S5C). Conversely, inhibiting autophagy—either by blocking autophagosome formation using 3-MA or chloroquine (CQ), or by preventing autophagosome degradation using the lysosomal inhibitor NH_4_Cl—led to the accumulation of SLC40A1 protein (Fig. S5D) and successfully impeded the TGF-β1-induced activation of autophagic flux (Fig. S5E). Functionally, such autophagy inhibition aggravated fibrosis (further increased α-SMA expression, Fig. S5F) and enhanced cellular resistance to ferroptosis (Fig. S5G). These findings suggest a mechanistic loop wherein SLC40A1 upregulation during fibrosis promotes fibroblast activation and inhibits ferroptosis; conversely, activated autophagy can degrade SLC40A1, thereby disrupting the ferroptosis resistance it mediates and consequently attenuating fibrotic progression.

### 2.5 SQSTM1 mediates the degradation of SLC40A1 via the selective autophagy pathway

Previous findings indicated that autophagy activation downregulates SLC40A1 protein. To elucidate the underlying regulatory mechanism, initial validation was performed in fibroblasts. Treatment with the autophagy inducers rapamycin (Rap), Cur, or under nutrient deprivation (EBSS) conditions effectively reduced SLC40A1 protein levels (Fig. 3A). Conversely, inhibition of autophagy led to the accumulation of SLC40A1 protein (Fig. 3B). Further protein stability assays demonstrated that the degradation rate of SLC40A1 was significantly accelerated under autophagy-activating conditions, a process that could be inhibited by NH_4_Cl (Fig. 3C). However, none of these treatments altered the mRNA level of SLC40A1 (Fig. 3D). Collectively, these data indicate that SLC40A1 is negatively regulated at the post-translational level through the autophagy-lysosomal pathway.

**Fig. 3:**
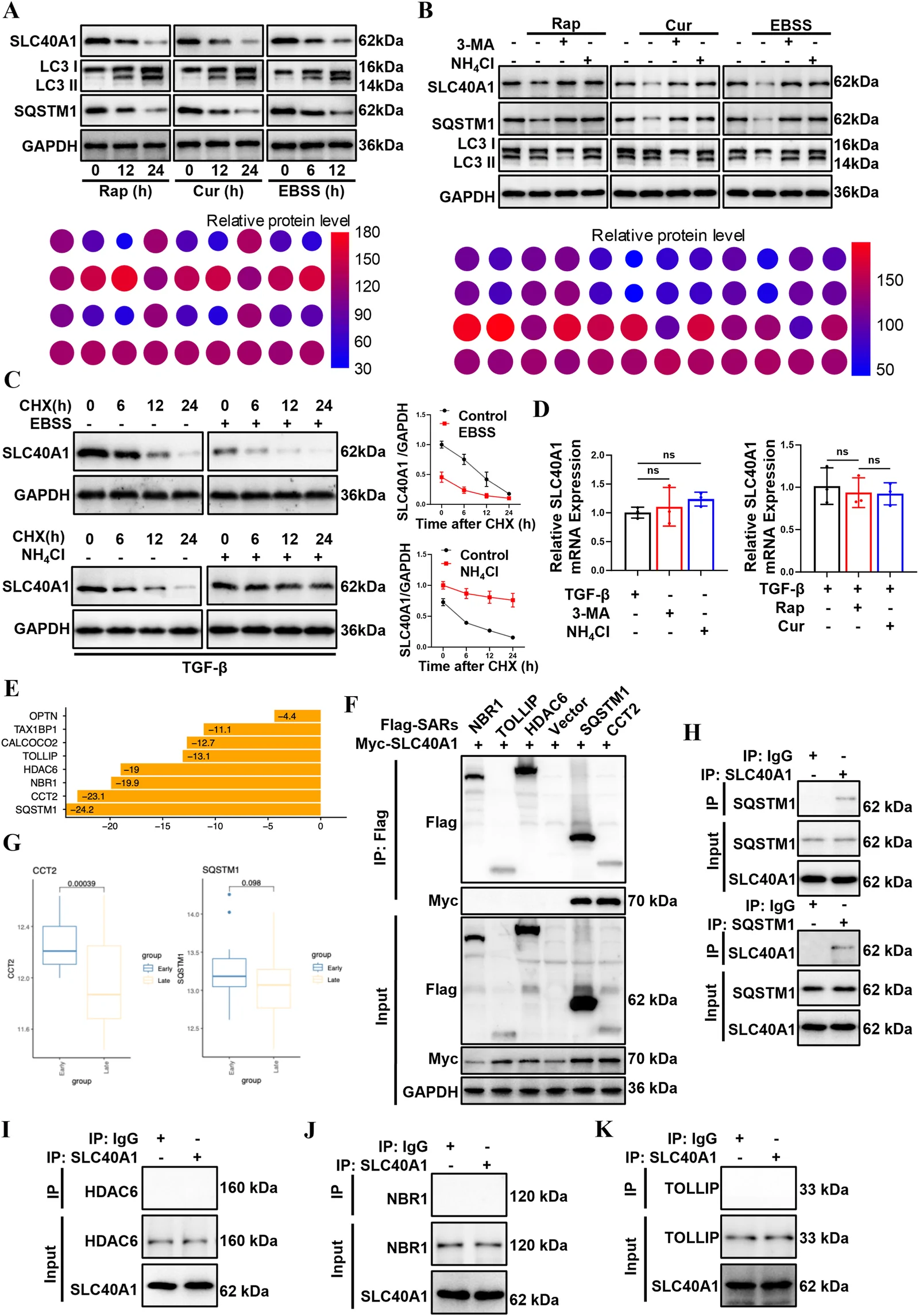
Autophagy regulates SLC40A1 protein stability through SQSTM1-mediated selective degradation. (A) Western blot analysis of SLC40A1 and autophagy marker proteins under treatment with autophagy activators. (B) Western blot analysis of SLC40A1 and autophagy marker proteins under treatment with autophagy inhibitors. (C) Western blot analysis of SLC40A1 protein degradation kinetics in a cycloheximide (CHX) chase assay. (D) qPCR analysis of SLC40A1 mRNA levels under the indicated treatments. (E) Heatmap showing the binding free energies (ΔG, kcal/mol) between SLC40A1 and eight selective autophagy receptors modeled by the AlphaFold Server and calculated using PRODIGY (v2.4.0). (F) Preliminary co-immunoprecipitation (co-IP) screening for interaction between SLC40A1 and selective autophagy receptors (SARs, including SQSTM1, CCT2, NBR1, TOLLIP, and HDAC6). (G) Analysis of SQSTM1 and CCT2 expression across different scar stages in public datasets. (H) Reciprocal co-immunoprecipitation validating the interaction between SQSTM1 and SLC40A1. (I-K) Co-immunoprecipitation results excluding interactions between SLC40A1 and other autophagy receptors (NBR1, TAX1BP1, OPTN). Data represent mean ± SD from n = 3 independent biological repeats. Western blot bands were quantified by densitometry using ImageJ. Statistical analyses were performed using one-way ANOVA with Tukey's multiple comparison test (ns = not significant).

We next aimed to identify the selective autophagy receptor mediating this process. Computational analysis of binding energies with eight known selective autophagy receptors (SARs) screened five potential candidates (Fig. 3E). Subsequent co-immunoprecipitation assays preliminarily suggested possible interactions between SLC40A1 and SQSTM1 or CCT2 (Fig. 3F). Analysis of public databases revealed that both CCT2 and SLC40A1 were significantly downregulated in late-stage scar tissues (Fig. 3G), consistent with a pattern of active degradation and suggesting that CCT2 might not be the key stable regulator of SLC40A1 under pathological conditions. Further reciprocal co-immunoprecipitation experiments conclusively confirmed a specific interaction between SQSTM1 and SLC40A1, while excluding interactions with other receptors (Fig. 3H–K).

To validate the functional role of SQSTM1, a series of experiments were conducted. First, TGF-β treatment attenuated the SQSTM1-SLC40A1 interaction, while the autophagy activator Rap enhanced this binding (Fig. S6A). Second, mutation of the ubiquitin-associated (UBA) domain in SQSTM1 (ΔUBA) completely abolished its interaction with SLC40A1 (Fig. S6B), led to significant accumulation of SLC40A1 protein, and attenuated its Rap-induced degradation (Fig. S6C). More critically, knockdown of SQSTM1 significantly delayed the degradation rate of SLC40A1 (Fig. S6D) and caused its protein accumulation (Fig. S6E). Conversely, overexpression of SQSTM1 promoted SLC40A1 degradation, an effect reversible by autophagy inhibitors (Fig. S6F). Finally, involvement of CCT2 was definitively excluded, as mutation of its enzymatic active site did not affect binding to SLC40A1 (Fig. S6G), and knockdown of CCT2 did not influence SLC40A1 protein levels (Fig. S6H). These results demonstrate that SQSTM1 functions as a selective autophagy receptor, recognizing and binding to SLC40A1 via its UBA domain, thereby targeting it to autolysosomes for degradation.

### 2.6 The E3 ubiquitin ligase NEURL1 promotes the autophagic degradation of SLC40A1 by catalyzing its K63-Linked ubiquitination

To elucidate the upstream regulatory mechanism targeting SLC40A1 for autophagic degradation, we focused on post-translational modifications. Protein-protein interaction network analysis identified the E3 ubiquitin ligase NEURL1 as a potential hub molecule (Fig. 4A). Further intersection analysis of scar progression datasets with an E3 ubiquitin ligase profile pinpointed NEURL1 as a key candidate factor (Fig. 4B). Clinical correlation analysis revealed a negative correlation between NEURL1 and SLC40A1 expression in scar tissues. Furthermore, NEURL1 expression correlated positively with ferroptosis susceptibility, whereas SLC40A1 showed an inverse correlation (Fig. S7A and B). Notably, NEURL1 mRNA levels were significantly downregulated in late-stage disease (Fig. 4C), consistent with its hypothesized role as a negative regulator of SLC40A1. Our ubiquitin ligase screening indicated that NEURL1 was the sole candidate that both interacted with SLC40A1 and was downregulated in scar tissue (Fig. 4A and B). Co-immunoprecipitation validation of other candidate E3 ligases, such as TRIM21, showed no detectable binding, thereby confirming the specificity of NEURL1.

**Fig. 4:**
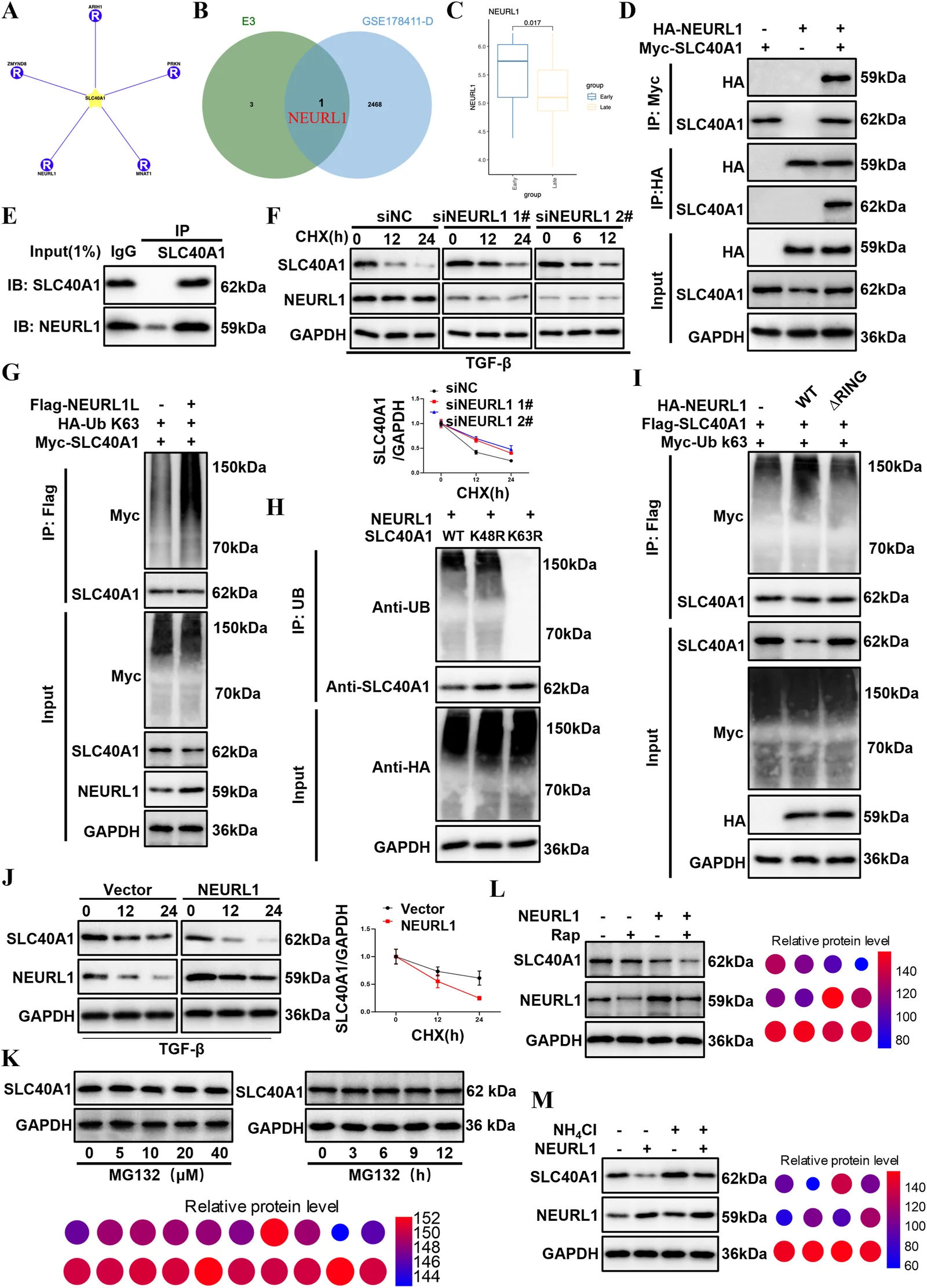
The E3 ligase NEURL1 targets SLC40A1 for autophagic-lysosomal degradation by catalyzing its K63-linked ubiquitination. (A) Protein-protein interaction network analysis highlighting NEURL1 as a central node. (B) Venn diagram analysis intersecting differentially expressed genes from dataset GSE178411 with an E3 ubiquitin ligase profile, identifying NEURL1 as a key candidate. (C) Box plot showing the mRNA expression level of NEURL1 across different stages of scar development. (D) Co-IP assay validating the interaction between NEURL1 and SLC40A1. (E) Endogenous co-IP assay validating the interaction between NEURL1 and SLC40A1 in scar tissues. (F) Western blot analysis of SLC40A1 protein degradation kinetics following NEURL1 knockdown. (G) Immunoprecipitation assay detecting the level of K63-linked ubiquitination on SLC40A1 in the presence of NEURL1. (H) Immunoprecipitation assay assessing the effect of K48R and K63R point mutations in SLC40A1 on its ubiquitination. (I) Immunoprecipitation assay evaluating the impact of a RING domain mutation in NEURL1 on its ability to catalyze K63-linked ubiquitination of SLC40A1. (J) Western blot analysis of SLC40A1 protein degradation kinetics upon NEURL1 overexpression. (K) Western blot analysis of SLC40A1 protein levels after treatment with the proteasome inhibitor MG132. (L) Western blot analysis of SLC40A1 protein levels upon NEURL1 overexpression combined with treatment with the autophagy activator rapamycin (Rap). (M) Western blot analysis of SLC40A1 protein levels upon NEURL1 overexpression combined with treatment with the lysosomal inhibitor NH_4_Cl. Data represent mean ± SD from n = 3 independent biological replicates. Statistical comparisons were made using two-way ANOVA followed by Tukey's post hoc test (*p < 0.05, **p < 0.01, ***p < 0.001).

Subsequent experiments confirmed a physical interaction between NEURL1 and SLC40A1 (Fig. 4D and E). Functionally, knockdown of NEURL1 significantly delayed the protein degradation rate of SLC40A1 (Fig. 4F), while its overexpression accelerated SLC40A1 degradation (Fig. 4J–S7E). However, neither knockdown nor overexpression of NEURL1 significantly altered SLC40A1 mRNA levels (Fig. S7C). We further investigated the type of ubiquitin linkage catalyzed by NEURL1. Immunoprecipitation assays demonstrated that NEURL1 markedly enhanced K63-linked polyubiquitination of SLC40A1 (Fig. 4G). Site-directed mutagenesis confirmed that lysine 63 (K63), but not lysine 48 (K48), on SLC40A1 was critical for its ubiquitination (Fig. 4H). Moreover, mutation of the RING domain in NEURL1 (essential for its catalytic activity) abolished K63-linked ubiquitination of SLC40A1 (Fig. 4I) and delayed its degradation (Fig. S7D), demonstrating the enzyme activity-dependence of NEURL1's function.

We next determined the degradation pathway through which NEURL1 directs SLC40A1. Treatment with the proteasome inhibitor MG132 did not cause SLC40A1 accumulation (Fig. 4K), ruling out the proteasomal pathway. Conversely, the autophagy inducer rapamycin (Rap) synergized with NEURL1 overexpression to further reduce SLC40A1 levels (Fig. 4L), whereas the lysosomal inhibitor NH_4_Cl completely blocked NEURL1-mediated SLC40A1 degradation (Fig. 4M), confirming the predominance of the autophagy-lysosomal pathway. Mechanistically, the presence of NEURL1 significantly enhanced the binding between SQSTM1 and SLC40A1 (Fig. S7G). Furthermore, the NEURL1-mediated downregulation of SLC40A1 was reversed upon SQSTM1 knockdown (Fig. S7H), indicating that NEURL1's function is dependent on SQSTM1. This regulatory process was dynamically modulated by pathological signals: TGF-β treatment weakened, while Rap treatment strengthened, the NEURL1-SLC40A1 interaction (Fig. S7F).

Moreover, as shown in Fig. S8A, we examined the effects of NEURL1 overexpression on ferroptosis-related indicators. Ectopic NEURL1 expression triggered pronounced ferroptotic stress in fibroblasts, characterized by accumulation of lipid hydroperoxides (Fig. S8B), elevated mitochondrial superoxide generation (Fig. S8C), and a surge in total intracellular ROS (Fig. S8D). Concomitantly, NEURL1 overexpression expanded the intracellular labile iron pool, accelerated MDA production, and depleted the antioxidant GSH reserve (Fig. S8E). Importantly, these pro-ferroptotic perturbations induced by NEURL1 were significantly attenuated by SLC40A1 co-overexpression and almost completely abrogated by the canonical ferroptosis inhibitor Ferrostatin-1 (Fig. S8B–E), demonstrating that NEURL1 triggers ferroptosis in fibroblasts through the downregulation of its downstream effector SLC40A1.

Collectively, these findings demonstrate that the E3 ubiquitin ligase NEURL1 catalyzes K63-linked polyubiquitination of SLC40A1. This modification enhances the recognition and binding of SLC40A1 by the selective autophagy receptor SQSTM1, thereby targeting it for degradation via the autolysosomal pathway.

### 2.7 Curcumin promotes ferroptosis and inhibits fibrosis by enhancing NEURL1-Mediated K63 ubiquitination and subsequent autophagic degradation of SLC40A1

Based on the aforementioned findings, we further investigated how pharmacological intervention finely tunes the NEURL1-SQSTM1-SLC40A1 degradation axis. We observed that treatment with the autophagy activator Cur significantly enhanced the total ubiquitination level of SLC40A1 (Fig. 5A) as well as its specific K63-linked ubiquitination (Fig. 5B and C). This process was accompanied by strengthened binding between SQSTM1 and SLC40A1 (Fig. 5D), suggesting that Cur may increase the efficiency of SLC40A1 recognition and capture by SQSTM1 via promoting its K63 ubiquitination.

**Fig. 5:**
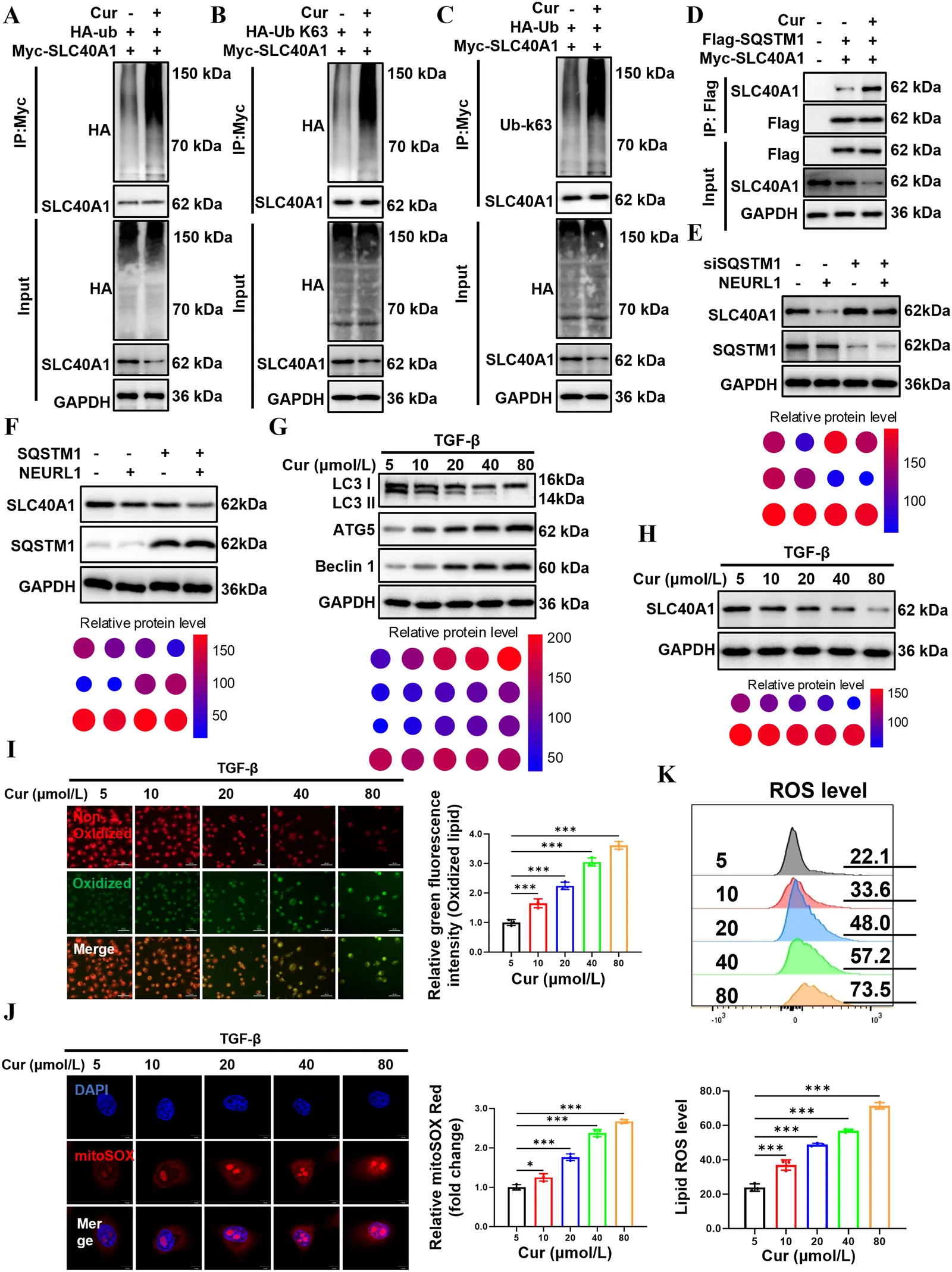
Curcumin promotes ferroptosis and suppresses fibrosis by enhancing NEURL1/SQSTM1-mediated K63 ubiquitination and autophagic degradation of SLC40A1. (A) Immunoprecipitation assay for total ubiquitination levels of SLC40A1 following curcumin (Cur) treatment. (B) Immunoprecipitation assay for specific K63-linked ubiquitination levels of SLC40A1 following Cur treatment. (C) Validation of K63-linked ubiquitination of SLC40A1 using an HA-ubiquitin pulldown assay and a K63 linkage-specific antibody. (D) Co-immunoprecipitation assay showing the effect of Cur treatment on the interaction between SQSTM1 and SLC40A1. (E) Western blot analysis showing the impact of SQSTM1 knockdown on the ability of NEURL1 overexpression to downregulate SLC40A1. (F) Western blot analysis of SLC40A1 protein levels upon co-overexpression of NEURL1 and SQSTM1. (G) Western blot analysis of autophagy-related proteins (LC3Ⅱ/Ⅰ, ATG5, Beclin 1) under treatment with different concentrations of Cur. (H) Western blot analysis of SLC40A1 protein expression under treatment with different concentrations of Cur. (I) Representative fluorescence microscopy images showing total intracellular ROS levels under treatment with different concentrations of Cur. (J) Representative confocal fluorescence microscopy images showing mitochondrial ROS levels under treatment with different concentrations of Cur. (K) Flow cytometry analysis of intracellular ROS levels. Data are shown as mean ± SD from n = 3 independent biological replicates. Fluorescence intensity in (I, J) was quantified from at least 5 random visual fields per condition using ImageJ. Statistical analysis was performed using one-way ANOVA with Tukey's post hoc test (*p < 0.05, **p < 0.01, ***p < 0.001).

To validate the synergistic role of NEURL1 and SQSTM1 in this pathway, functional complementation experiments were performed. Results showed that knockdown of SQSTM1 blocked the downregulatory effect of NEURL1 overexpression on SLC40A1 (Fig. 5E), whereas co-overexpression of both produced a stronger degradative effect (Fig. 5F). This confirms that NEURL1-catalyzed K63 ubiquitination serves as a crucial prerequisite for the subsequent SQSTM1-mediated autophagic degradation, together constituting a coherent “tag-and-recognize” cascade.

Subsequently, we systematically evaluated the dose-dependent effects of Cur in a TGF-β-induced fibroblast model. As expected, Cur activated autophagy in a dose-dependent manner (Fig. 5G) and concurrently downregulated SLC40A1 protein levels (Fig. 5H). Functionally, Cur treatment effectively promoted ferroptosis, manifested by a dose-dependent and significant increase in both intracellular and mitochondrial ROS levels (Fig. 5I–K). Simultaneously, Cur also inhibited fibrotic phenotypes in a dose-dependent manner, including fibroblast migration (Fig. S9A and C), myofibroblast transdifferentiation (Fig. S9B), extracellular matrix synthesis, and the expression of matrix remodeling proteins (Fig. S9D and E). To investigate whether NEURL1 mediates the induction of ferroptosis by Cur in an E3 ligase activity-dependent manner, we compared the effects of NEURL1-WT and a RING domain deletion mutant lacking ubiquitin ligase activity NEURL1-ΔRING in the presence of Cur (Fig. S9F). Cur treatment provoked lipid peroxidation (Fig. S9G), mitochondrial superoxide overproduction (Fig. S9H), and total intracellular ROS surge (Fig. S9I) in wild-type NEURL1 (NEURL1-WT)-expressing fibroblasts; however, these oxidative events were substantially blunted in cells expressing the catalytically inactive RING-deletion mutant (NEURL1-ΔRING). Concordantly, Cur-induced labile iron accumulation, MDA elevation, and GSH depletion were largely refractory to Cur in NEURL1-ΔRING cells (Fig. S9J), demonstrating that NEURL1-catalyzed ubiquitination is indispensable for the activation of ferroptosis by Cur.

In summary, these findings elucidate that Cur exerts its multi-faceted anti-fibrotic effects—downregulating SLC40A1, activating ferroptosis, and inhibiting fibroblast activation—by enhancing NEURL1-mediated K63 ubiquitination of SLC40A1, thereby potentiating its SQSTM1-dependent selective autophagic degradation.

### 2.8 A microneedle-mediated Co-delivery system for curcumin and siSLC40A1 synergistically inhibits scarring

To achieve targeted transdermal intervention, we engineered a dissolvable microneedle system co-delivering exosome-encapsulated curcumin and SLC40A1-targeting siRNA (MNs@Exo@siSLC40A1@Cur, termed Complex) for hypertrophic scar treatment in vivo (Fig. 6A). The Complex displayed superior therapeutic efficacy, yielding the most prominent scar flattening, minimal dermal thickness, and well-aligned collagen architecture (Fig. 6C and D). At the molecular level, topical Complex delivery achieved profound ablation of SLC40A1 protein (Fig. 6B, Fig. S10A), robustly suppressed myofibroblast activation (Fig. 6E, Fig. S10C), and curtailed pathological angiogenesis (Fig. 6G, Fig. S10B). Notably, head-to-head comparison confirmed that microneedle-assisted transdermal delivery achieved substantially greater anti-fibrotic potency and tissue remodeling than topical administration of the unencapsulated free-drug combination (Fig. S10D–I), underscoring the necessity of the microneedle delivery platform for overcoming the dense scar stratum corneum barrier.

**Fig. 6:**
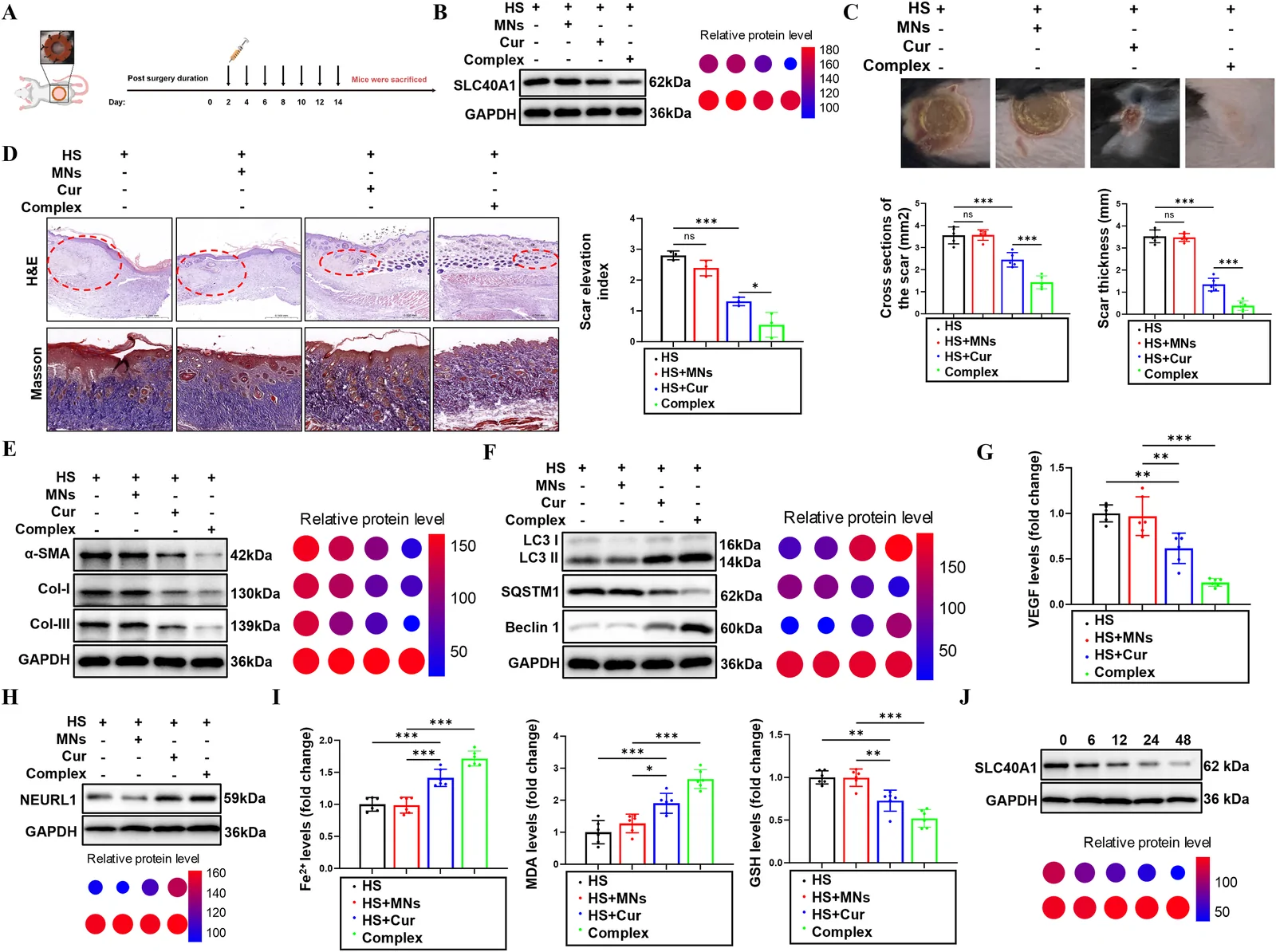
Microneedle-mediated co-delivery of curcumin and siSLC40A1 alleviates pathological scarring by activating autophagy and inhibiting angiogenesis. (A) Schematic diagram of the mouse scar model and the microneedle-based treatment strategy. (B) Western blot analysis of SLC40A1 protein expression in scar tissues from each treatment group. (C) Representative macroscopic photographs of dorsal scars from each group and quantitative analysis of the scar index. (D) Representative images of scar tissue sections stained with Hematoxylin and Eosin (H&E) and Masson's trichrome. (E) Western blot analysis of fibrotic marker proteins (α-SMA, Col-I, Col-III). (F) Western blot analysis of autophagic flux marker proteins (LC3Ⅱ/Ⅰ, Beclin 1, SQSTM1). (G) Quantitative analysis of vascular endothelial growth factor (VEGF) levels in tissues. (H) Western blot analysis showing the expression levels of NEURL1 protein. (I) Measurement of ferroptosis-related biomarkers: intracellular Fe^2+^, malondialdehyde (MDA), and glutathione (GSH) levels. (J) Time-course Western blot analysis of SLC40A1 protein degradation following treatment exposure (0, 6, 12, 24, 48 h). Complex means MNs@Exo@siSLC40A1@Cur Data are presented as mean ± SD (n = 6 mice per group). For histological quantification in (C, D), 5 random non-overlapping fields per tissue section were analyzed using ImageJ. Statistical significance was assessed using one-way ANOVA followed by Tukey's post hoc test (*p < 0.05, **p < 0.01, ***p < 0.001).

Mechanistically, the Complex simultaneously triggered autophagic flux and targeted SLC40A1 for degradation in vivo (Fig. 6F). Microneedle intervention markedly upregulated NEURL1 while expediting SQSTM1-dependent clearance of SLC40A1, with progressive depletion observed over 48 h (Fig. 6H–J). Consequently, targeted SLC40A1 downregulation broke the ferroptosis resistance in scar tissues, provoking significant labile iron accumulation, MDA elevation, and concomitant GSH depletion (Fig. 6I). These i*n vivo* findings corroborate that activating the NEURL1–SQSTM1–SLC40A1 axis effectively eradicates activated fibroblasts by unleashing ferroptosis.

Comprehensive safety evaluations substantiated the translational biocompatibility of the microneedle formulation. Complex-treated mice exhibited intact histomorphology across major visceral organs (Fig. S11A), stable body weight gain, and normal hepatic and renal function profiles (Fig. S11B). Furthermore, microneedle application elicited no local epidermal cytotoxicity (Fig. S11D) and caused no significant elevation of cutaneous pro-inflammatory cytokines (Fig. S11C), confirming the favorable biosafety profile of this transdermal therapeutic regimen.

## 3 Discussion

This study clarifies that the iron exporter SLC40A1 is highly expressed in pathological scars and serves as a key factor enabling fibroblasts to acquire ferroptosis resistance and sustain activation. We demonstrate for the first time that the stability of SLC40A1 is regulated by a novel selective autophagy pathway, which is sequentially mediated by the E3 ubiquitin ligase NEURL1 and the autophagy receptor SQSTM1. Both in vivo and in vitro experiments confirm that activating this “NEURL1-SQSTM1-SLC40A1” degradation axis through pharmacological or genetic approaches can effectively induce ferroptosis in fibroblasts and significantly inhibit scar formation.

The reliability of our findings is reinforced by their alignment with well-established regulatory paradigms in cell biology and the multi-layered, mutually corroborative evidence presented. First, as a key iron exporter, the expression level of SLC40A1 directly influences the size of the intracellular labile iron pool, which constitutes the core biochemical determinant of ferroptosis susceptibility [16,[28], [29], [30]]. The observed association in this study between high SLC40A1 expression, low lipid peroxidation, and high ferroptosis resistance is entirely consistent with this widely validated theoretical framework of ferroptosis regulation. Second, SQSTM1 is a classical selective autophagy receptor whose function depends on the recognition of ubiquitinated substrates [[31], [32], [33], [34], [35], [36]]. Our finding—that SQSTM1 recognizes and degrades the ubiquitinated SLC40A1—represents a specific manifestation of this general mechanism within a particular pathological context, demonstrating intrinsic logical consistency.

More critically, our expansion of NEURL1's function is supported by sound biochemical principles. While previous studies have primarily established NEURL1's ability to catalyze K48-linked ubiquitin chains for proteasomal degradation, it is not uncommon for an E3 ligase to catalyze different ubiquitin linkage types [[37], [38], [39], [40]]; its specificity is often dictated by the substrate, cellular context, and co-factors. In this study, after rigorously excluding a major role for K48-linkage, we confirmed NEURL1-mediated K63 ubiquitination of SLC40A1. This finding does not contradict the known mechanism but rather reveals the context-dependent functionality of NEURL1. K63-linked ubiquitination typically participates in signal transduction and autophagic degradation [[41], [42], [43], [44]]. Our results therefore logically bridge NEURL1's catalytic activity with the SQSTM1-mediated autophagy pathway, forming a complete regulatory cascade that fits seamlessly within the existing knowledge framework of cellular signaling.

Traditionally, SLC40A1 is regarded as a gatekeeper maintaining iron homeostasis [30,45]. However, our findings in the scar microenvironment ascribe to it a novel pathological significance: highly expressed SLC40A1, by continuously pumping iron out of the cell, artificially creates an intracellular “low-iron” milieu. This directly counteracts the core trigger of ferroptosis—iron-dependent lipid peroxidation. This “iron efflux advantage” enables myofibroblasts to evade ferroptosis, a crucial cell clearance program, thereby acquiring long-term survival and persistent collagen secretion capacity, which drives scar hyperplasia. This discovery provides a fresh perspective for understanding the persistence of effector cells in fibrotic diseases.

Furthermore, our work establishes a clear, mechanistic link between autophagy activation and ferroptosis induction through a sequential molecular axis: NEURL1–SQSTM1–SLC40A1. The identification of this quantitative regulatory pathway offers a new explanation for the seemingly paradoxical role of autophagy in fibrosis, which can be either protective or promoting. It suggests that autophagy may alter a cell's death threshold by selectively degrading specific pro-survival proteins, such as SLC40A1.

Within the field of ubiquitination, our work provides a significant addition. As mentioned, we present the first experimental evidence that NEURL1 catalyzes K63-linked ubiquitination, leading to the subsequent autophagic degradation of its substrate. This finding challenges the traditional view of NEURL1 functioning solely via K48-linkage in proteasomal degradation [[46], [47], [48]], broadening the understanding of its biological functions and serving as a potential paradigm for investigating similar “functional pleiotropy” in other E3 ligases.

In comparison with existing research in the field, our results align with the broader hypothesis that “inhibition of ferroptosis promotes fibrosis.” However, our study concretely specifies and mechanistically elucidates this concept in the context of skin scarring. Unlike research in hepatic or pulmonary fibrosis that often focuses on antioxidant systems [49,50], we targeted the “export” regulation of iron metabolism. This approach revealed tissue- or cell type-specific regulatory nodes, thereby enriching the map of regulatory networks that encompass both common and distinctive features across organ fibroses.

This study holds dual implications for clinical translation. First, the expression levels of SLC40A1 and its upstream regulators may serve as potential molecular biomarkers for assessing the biological activity and prognosis of scars. More importantly, we have designed and validated an innovative therapeutic strategy based on this mechanism. We constructed a microneedle-based transdermal delivery system co-loaded with the autophagy activator curcumin (Cur) and exosomes (Exo) encapsulating siSLC40A1, along with permeation-enhancing components [[51], [52], [53]]. This design ingeniously achieves synergistic targeted delivery: curcumin activates the autophagy pathway upstream, while siSLC40A1 directly knocks down the target protein downstream. Both agents act through the same mechanistic axis we elucidated, thereby maximizing the spatiotemporal activation of the “NEURL1-SQSTM1-SLC40A1” degradation cascade. Animal studies confirmed that this system potently induces local fibroblast ferroptosis, significantly inhibits scar hyperplasia, and exhibits no systemic toxicity. This approach provides a highly translatable formulation strategy addressing traditional challenges in anti-fibrotic therapy, such as inadequate local drug concentration, poor targeting, and systemic side effects.

This study has certain limitations. First, the upstream transcriptional mechanism responsible for NEURL1 downregulation in late-stage scars remains unclear; for instance, whether it is suppressed by pathways such as TGF-β/Smad. Future studies should identify the relevant transcription factors and explore whether small-molecule agonists can restore NEURL1 function. Second, the long-term stability and release profile of exosomes within the microneedle system require further optimization, including adjustment of the HA matrix degradation rate and encapsulation efficiency. Third, the clinical sample size was limited; expanding the cohort will be necessary to validate SLC40A1 as a biomarker of scar activity and to strengthen the clinical relevance of the findings [[54], [55], [56]]. Concurrently, systematic evaluation of the dosing regimen, long-term efficacy, and immunogenicity of the microneedle combination therapy in large animal models that more closely mimic human skin pathophysiology will be crucial steps toward clinical translation.

## 4 Materials and methods

### 4.1 Animal models and in vivo treatment

Male C57BL/6 mice (8-week-old) were used to establish hypertrophic scar models. Two methods were employed: 1) A mechanical load-induced model, where a dorsal incision was subjected to sustained tension using a distraction device; 2) A silicone ring splint model, where an excisional wound was mechanically stretched. Scar tissues were harvested at the proliferative phase (day 3 post-wounding) and the mature phase (after 2 weeks). For therapeutic intervention, mice received subcutaneous injections of either curcumin (Cur, 2 mM) or the ferroptosis inducer RSL3 (50 mg/kg every 3 days for 21 days) around the wound. All procedures were approved by the Institutional Animal Care and Use Committee No. FE20002.

### 4.2 Cell culture and treatment

Primary human dermal fibroblasts from normal skin (NSF) and hypertrophic scars (HSF) were cultured in DMEM supplemented with 10% FBS and 1% penicillin–streptomycin at 37°C with 5% CO_2_. Fibroblast activation was induced by treatment with recombinant human TGF-β1 (10 ng/mL, PeproTech) for 48 h, unless otherwise specified. This concentration was selected as the optimal activation dose based on preliminary concentration-gradient experiments. For pharmacological interventions, cells were treated with curcumin (Cur, 0–80 μM, Med Chem Express), RSL3 (1 μM, Selleck), Rapamycin (Rap, 100 nM, Selleck), 3-Methyladenine (3-MA, 5 mM, Selleck), Chloroquine (CQ, 20 μM, Sigma), NH_4_Cl (20 mM, Sigma), or Ferrostatin-1 (Fer-1, 1 μM, Selleck) for the indicated durations.

### 4.3 Plasmid transfection and RNA interference

Overexpression plasmids for SLC40A1, NEURL1, SQSTM1, and their respective mutants (SLC40A1-K63R, NEURL1-ΔRING, SQSTM1-ΔUBA) were constructed. Small interfering RNAs (siRNAs) targeting SLC40A1, NEURL1, SQSTM1, CCT2, and corresponding negative controls (siNC) were purchased from GenePharma. Transfections were performed using Lipofectamine 3000 (Invitrogen) according to the manufacturer's protocol.

### 4.4 Western blotting

Total protein was extracted from tissues or cells using RIPA buffer containing protease and phosphatase inhibitors. Protein concentration was determined via BCA assay. Equal amounts of protein were separated by SDS-PAGE, transferred to PVDF membranes, and blocked with 5% non-fat milk. Membranes were incubated overnight at 4°C with primary antibodies against SLC40A1, α-SMA, COL-I, COL-III, LC3Ⅱ/Ⅰ, SQSTM1, Beclin 1, ATG5, GPX4, SLC7A11, TFRC, FTH1, NEURL1, SQSTM1, CCT2, and GAPDH, followed by incubation with HRP-conjugated secondary antibodies. Bands were visualized using enhanced chemiluminescence (ECL).

### 4.5 Quantitative real-time PCR (qPCR)

Total RNA was extracted using TRIzol reagent and reverse transcribed into cDNA. qPCR was performed using SYBR Green Master Mix on a QuantStudio system. The mRNA levels of target genes were normalized to GAPDH and calculated using the 2^–ΔΔCt method.

### 4.6 Immunofluorescence and immunohistochemistry

For immunofluorescence, cells or tissue sections were fixed, permeabilized, blocked, and incubated with primary antibodies against SLC40A1, α-SMA, or CD31 overnight at 4°C, followed by incubation with fluorescent secondary antibodies and DAPI. Images were captured using a confocal microscope. For immunohistochemistry, paraffin-embedded tissue sections were stained with antibodies against α-SMA and CD31, visualized with DAB, and counterstained with hematoxylin.

#### 4.6.1 Immunoprecipitation and ubiquitination assay

For Co-IP, cell lysates were incubated with specific antibodies or control IgG overnight at 4°C, followed by incubation with Protein A/G agarose beads. The precipitated complexes were washed and analyzed by Western blot. For denatured immunoprecipitation, cell lysates were boiled in TBS containing 1% SDS and 10 mM N-ethylmaleimide (NEM) at 95°C for 10 min to disrupt non-covalent protein–protein interactions. The denatured lysates were subsequently diluted 10-fold with SDS-free lysis buffer to reduce the final SDS concentration to 0.1% prior to antibody incubation and immunoprecipitation. Bound proteins were eluted by boiling in 2× Laemmli buffer and analyzed by western blotting with anti-Myc, anti-HA, or linkage-specific ubiquitin antibodies. For ubiquitination assays, cells were co-transfected with HA-Ubiquitin (or HA-Ub-K63/K48 only) and target plasmids, treated with MG132 (10 μM) for 6 h before harvest. Cell lysates were subjected to IP with anti-SLC40A1 antibody, and the ubiquitination level was detected using anti-HA or anti-K63-linkage specific antibodies.

### 4.7 CHX chase assay

Cells were treated with cycloheximide (CHX, 100 μg/mL) to inhibit protein synthesis and harvested at indicated time points. The degradation rate of SLC40A1 protein was assessed by western blotting.

### 4.8 Biochemical assays

Malondialdehyde (MDA) and glutathione (GSH) levels were measured using commercial assay kits (Nanjing Jiancheng Bioengineering Institute). Intracellular Fe^2+^ levels were quantified using an Iron Assay Kit (Abcam). Reactive oxygen species (ROS) were detected using DCFH-DA probe (Beyotime), and mitochondrial ROS was detected using MitoSOX Red (Invitrogen), following manufacturers' protocols.

### 4.9 Cell viability, migration, and proliferation assays

Cell viability was assessed by CCK-8 assay. Cell proliferation was monitored using CFDA-SE staining and flow cytometry. Cell migration was evaluated by wound healing (scratch) assay and Transwell chamber assay.

### 4.10 Bioinformatic analysis

Gene expression datasets GSE72901 and GSE178411 were downloaded from the Gene Expression Omnibus (GEO) database. Differential gene expression analysis was performed using the limma package in R. Venn diagrams were generated to identify intersecting genes. Functional enrichment analysis was conducted using clusterProfiler. Protein-protein interaction network analysis and E3 ligase screening were performed using the STRING database and published ubiquitin ligase libraries.

### 4.11 Histological and safety evaluation

Scar and major organ tissues were fixed, embedded, sectioned, and stained with Hematoxylin & Eosin (H&E) or Masson's trichrome. Serum biochemical parameters (ALT, AST, UREA, CREA) were measured using an automatic biochemical analyzer.

### 4.12 Design and fabrication of dissolvable microneedle (MN) patches

Dissolvable microneedle (MN) patches were fabricated via a mold-based method using hyaluronic acid (HA, MW = 1.2 × 10^6^ Da) as the primary matrix material. An aqueous HA solution (20 wt%) was cast into polydimethylsiloxane molds with conical cavities (needle length: 500 μm, base diameter: 100 μm). After vacuum-assisted drying and demolding, the resulting blank HA MN arrays provided a painless, efficient platform for transdermal delivery, capable of penetrating dense scar tissue. For active loading, curcumin (Cur, 20 μM) was encapsulated into exosomes (Exo) isolated from mouse fibroblasts using a thin-film hydration method, forming Exo-Cur. Separately, siRNA targeting SLC40A1 (siSLC40A1) was loaded into exosomes via electroporation to yield Exo@siSLC40A1. The two exosome populations were then mixed to obtain the co-loaded composite (Exo-Cur@siSLC40A1). This composite was incorporated into the HA matrix during the casting process. The final drug loading efficiency was determined to be 89.2 ± 3.1% for Cur and 78.5 ± 2.7% for siSLC40A1, as quantified by HPLC and fluorescence-based RNA assay, respectively.

#### 4.12.1 Protein complex modeling and binding affinity calculation

The canonical amino acid sequences of human SLC40A1 (UniProt ID: Q9NP59) and eight candidate selective autophagy receptors—CALCOCO2 (UniProt ID: Q13137), CCT2 (UniProt ID: P78371), HDAC6 (UniProt ID: Q9UBN7), NBR1 (UniProt ID: Q14596), OPTN (UniProt ID: Q96CV9), SQSTM1 (UniProt ID: Q13501), TAX1BP1 (UniProt ID: Q86VP1), and TOLLIP (UniProt ID: Q9H0E2)—were retrieved from the UniProt database. The structural models of binary protein complexes between SLC40A1 and each receptor were predicted using the AlphaFold Server (https://alphafoldserver.com/) using default parameter settings. The binding free energies (ΔG, kcal/mol) and dissociation constants were calculated using PRODIGY (PROtein binDIng enerGY prediction, version 2.4.0). The calculated binding energy heatmap was visualized using the ggplot2 package (version 4.0.3) in R.

### 4.13 Statistical analysis

Data are presented as mean ± standard deviation (SD) from at least three independent experiments. The normality of data distribution was confirmed using the Shapiro-Wilk test, and homogeneity of variances was verified with Levene's test, confirming that the data met the assumptions for parametric tests. Statistical analyses were performed using GraphPad Prism 9.0 software. Differences between two groups were analyzed by Student's t-test (two-tailed). Comparisons among multiple groups were conducted using one-way or two-way analysis of variance (ANOVA) followed by Tukey's post hoc test for multiple comparisons. A p-value < 0.05 was considered statistically significant.

## CRediT authorship contribution statement

**Mengzhu Lv:** Writing – original draft, Validation, Investigation, Formal analysis. **Jiamei Liu:** Writing – original draft, Validation, Investigation, Formal analysis. **Qi Dong:** Writing – original draft, Validation, Investigation, Formal analysis. **Zibo Guo:** Investigation, Formal analysis. **Qianlong Wu:** Investigation, Formal analysis. **Simeng Zhang:** Writing – review & editing, Validation, Methodology, Conceptualization. **Shengye Liu:** Writing – review & editing, Validation, Methodology, Conceptualization.

## Consent to participate

Not applicable.

## Ethics approval

This study was approved by the Ethics committee of Shengjing Hospital of China Medical University.

## Consent for publication

Not applicable.

## Funding

This work was supported by Liaoning Province Science and Technology Plan Joint Program (Natural Science Foundation - General Project) (No. 2024-MSLH-579) and Collaborative Research Project of China Medical University (No. 30C&30D).

## Declaration of competing interest

The authors declare that they have no known competing financial interests or personal relationships that could have appeared to influence the work reported in this paper.

## Data Availability

The data analyzed during the current study are available from the corresponding author on reasonable request.
