# Supplementary material for: NEURL1-mediated K63-ubiquitination of SLC40A1 drives ferroptosis resistance in pathological scars: Therapeutic targeting via autophagy-activating microneedles
Source: Redox Biol. 2026 Sep 29;97:104420. doi: 10.1016/j.redox.2026.104420 (PMC13678046; doi:10.1016/j.redox.2026.104420)
Supplement: Supplementary file 1 [file mmc1.docx]

**Supplementary Figures**

Supplementary Figure 1. Bioinformatic analysis and clinical validation implicate SLC40A1 upregulation in ferroptosis resistance during pathological scar progression.

(A) Volcano plot of differentially expressed genes (DEGs) in the GSE72901 dataset (low glucose/autophagy activation model).

(B) Volcano plot of DEGs in the GSE178411 dataset (late-stage vs. early-stage scars).

(C) Venn diagram intersecting DEGs from (A), (B), and a ferroptosis inhibition gene set, identifying SLC40A1 as a key candidate.

(D, E) Functional enrichment analysis of the intersecting DEGs, highlighting associations with iron metabolism and oxidative stress pathways.

(F) Validation of SLC40A1 expression across the two datasets, showing downregulated expression under autophagy activation and upregulated expression during scar progression.

(G) Bioinformatics assessment comparing the ferroptosis score between early and late wound tissues.

(H) Relative mRNA expression level of SLC40A1 in clinical keloid scar specimens compared to normal skin tissues (n=20).

(I) Quantitative analysis of tissue iron deposition (n=6).

(J) Quantitative measurement of relative ferrous iron (Fe^2+^), ferric iron (Fe^3+^), and total iron levels in tissues (n=6).

(K) Measurement of relative MDA levels in clinical tissues (n=6).

(L) Western blot analysis (left) and corresponding quantitative bubble heatmap (right) of key ferroptosis-related proteins (n=3).

Data are presented as mean ± SD. Statistical differences between two groups were determined using two-tailed unpaired Student’s t-test (**p*<0.05, ***p*<0.01, ****p*<0.001).

Supplementary Figure 2. Curcumin and RSL3 remodel the pathological scar microenvironment by downregulating SLC40A1 and suppressing angiogenesis and fibroblast activation.

(A) Representative immunofluorescence staining images showing SLC40A1 protein (red) in tissue sections.

(B) Representative immunofluorescence staining images for CD31 (a vascular marker) and α-SMA (a myofibroblast marker) in tissue sections.

(C) Quantitative analysis of vascular endothelial growth factor (VEGF) levels in tissues.

Data represent mean ± SD (n=6 mice per group). For immunofluorescence signal quantification in (A, B), at least 5 randomly selected non-overlapping visual fields per section were analyzed blindly using ImageJ. One-way ANOVA followed by Tukey’s post hoc test was used for statistical comparison (**p*<0.05, ***p*<0.01, ****p*<0.001).

Supplementary Figure 3. Knockdown of SLC40A1 suppresses fibrotic phenotypes in fibroblasts by inducing ferroptosis.

(A) Cell migratory capacity assessed by wound healing (scratch) assay.

(B) Cell migratory capacity assessed by Transwell migration assay.

(C) Immunofluorescence staining of α-SMA demonstrating the activation status of fibroblasts.

(D) Western blot analysis of MMP2 and MMP9 protein expression.

(E) Western blot analysis of fibrotic marker protein expression (COL-I, COL-III, α-SMA).

Data are shown as mean ± SD from n=3 independent experiments. For cell migration and immunofluorescence in (A–C), at least 5 random fields per well/section were quantified using ImageJ. Statistical analysis was performed using one-way ANOVA followed by Tukey’s post hoc test (**p*<0.05, ***p*<0.01, ****p*<0.001).

Supplementary Figure 4. SLC40A1 Overexpression Promotes Fibroblast Activation and Ferroptosis Resistance, Which Is Reversed by Autophagy Activation.

(A) Bar graph comparing cell viability across different experimental groups assessed by CCK-8 assay.

(B) Proportion of viable cells as determined by CFDA-SE staining.

(C) Representative fluorescence images of intracellular oxidative stress detected by a redox-sensitive probe.

(D) Western blot analysis of SLC40A1 protein expression in the indicated groups.

(E) Levels of ferroptosis-related indicators (Fe²⁺, MDA, GSH).

(F) Cell migration assessed by Transwell assay (quantified cell counts).

(G) Immunofluorescence staining of α-SMA.

(H) Cell migratory capacity assessed by wound healing (scratch) assay.

Data are expressed as mean ± SD from n=3 independent biological replicates. Image quantification in (B, C, F, G, H) was performed across 5 random fields per group using ImageJ. One-way ANOVA with Tukey’s post hoc test was applied (**p*<0.05, ***p*<0.01, ****p*<0.001).

Supplementary Figure 5. Autophagy inhibition exacerbates fibroblast fibrosis and enhances ferroptosis resistance by upregulating SLC40A1.

(A) Western blot analysis of MMP2 and MMP9 protein expression.

(B) Western blot analysis of fibrotic marker proteins (COL-I, COL-III, α-SMA).

(C) Western blot analysis of autophagy-related proteins (LC3Ⅱ/Ⅰ, Beclin 1, ATG5, SQSTM1).

(D) Western blot analysis of SLC40A1 protein levels following treatment with autophagy inhibitors.

(E) Western blot analysis showing the impact of autophagy inhibitors on autophagic flux markers (LC3Ⅱ/Ⅰ, SQSTM1).

(F) Immunofluorescence staining of α-SMA after treatment with autophagy inhibitors.

(G) Effect of autophagy inhibitors on ferroptosis-related indicators (Fe²⁺, MDA, GSH).

Data represent mean ± SD from n=3 independent biological repeats. Signal intensities were analyzed by ImageJ. Statistical significance was determined using one-way ANOVA followed by Tukey’s post hoc test (**p*<0.05, ***p*<0.01, ****p*<0.001).

Supplementary Figure 6. SQSTM1 Mediates the Selective Autophagic Degradation of SLC40A1 via Its Ubiquitin-Associated (UBA) Domain.

(A) Co-immunoprecipitation (co-IP) assay showing the effect of TGF-β and Rap treatment on the SQSTM1-SLC40A1 interaction.

(B) Co-IP assay assessing the interaction between SLC40A1 and the SQSTM1 mutant lacking the UBA domain (ΔUBA).

(C) Western blot analysis of SLC40A1 protein levels in the presence of the SQSTM1 ΔUBA mutant.

(D) Western blot analysis of SLC40A1 protein degradation kinetics following SQSTM1 knockdown.

(E) Western blot analysis of SLC40A1 protein levels after SQSTM1 knockdown and Rap treatment.

(F) Western blot analysis of SLC40A1 protein levels after SQSTM1 overexpression and treatment with autophagy inhibitors.

(G) Co-IP assay assessing the interaction between SLC40A1 and a CCT2 mutant with a disrupted enzymatic active site.

(H) Western blot analysis of SLC40A1 protein levels following CCT2 knockdown.

Data are presented as mean ± SD from n=3 independent biological experiments. Western blot band densities were quantified using ImageJ. Statistical analysis was performed using one-way ANOVA or two-way ANOVA with Tukey’s post hoc test (**p*<0.05, ***p*<0.01, ****p*<0.001).

Supplementary Figure 7. NEURL1-mediated K63 ubiquitination of SLC40A1 is essential for its recognition by SQSTM1 and subsequent autophagic degradation.

(A) Scatter plot showing the correlation between NEURL1 and SLC40A1 expression levels in scar tissues.

(B) Correlation network depicting the relationships among NEURL1, SLC40A1, and various modes of cell death.

(C) RT-qPCR analysis of SLC40A1 and NEURL1 mRNA levels following NEURL1 knockdown (siNEURL1#1, siNEURL1#2) or overexpression (NEURL1).

(D) Western blot analysis of SLC40A1 protein degradation kinetics following mutation of the RING domain in NEURL1.

(E) Western blot analysis of SLC40A1 protein levels upon NEURL1 overexpression.

(F) Co-immunoprecipitation (co-IP) assay showing the effect of TGF-β and Rap treatment on the NEURL1-SLC40A1 interaction.

(G) Co-IP assay assessing the effect of NEURL1 on the SQSTM1-SLC40A1 interaction.

(H) Western blot analysis showing the impact of SQSTM1 knockdown on NEURL1-mediated downregulation of SLC40A1.

Data are expressed as mean ± SD from n=3 independent biological replicates. Statistical comparisons were performed using one-way or two-way ANOVA followed by Tukey’s post hoc test (ns = not significant, **p*<0.05, ***p*<0.01, ****p*<0.001).

Supplementary Figure 8. NEURL1 overexpression induces ferroptosis, which is rescued by SLC40A1 co‑overexpression or the ferroptosis inhibitor Ferrostatin‑1.

(A) Schematic of experimental groups.

(B) Assessment of lipid peroxidation by BODIPY™ 581/591 C11 staining.

(C) Measurement of mitochondrial superoxide levels by MitoSOX™ Red fluorescence.

(D) Flow cytometric analysis of intracellular ROS levels using DCFH‑DA probe.

(E) Quantification of ferroptosis‑related biochemical markers.

Data represent mean ± SD from n=3 independent biological replicates. Fluorescence images in (B, C) were quantified across 5 random visual fields per group using ImageJ. Statistical analysis was performed using one-way ANOVA with Tukey’s post hoc test (**p*<0.05, ***p*<0.01, ****p*<0.001).

Supplementary Figure 9. Curcumin inhibits TGF-β-induced fibroblast migration, activation, and extracellular matrix remodeling in a dose-dependent manner.

(A) Cell migration assessed by Transwell assay under treatment with different concentrations of curcumin.

(B) Immunofluorescence staining of α-SMA under treatment with different concentrations of curcumin.

(C) Cell migratory capacity assessed by wound healing (scratch) assay under treatment with different concentrations of curcumin.

(D) Western blot analysis of MMP2 and MMP9 protein expression under treatment with different concentrations of curcumin.

(E) Western blot analysis of fibrotic marker proteins (COL-I, COL-III, α-SMA) under treatment with different concentrations of curcumin.

(F) Schematic of experimental groups.

(G) Assessment of lipid peroxidation by BODIPY™ 581/591 C11 staining.

(H) Measurement of mitochondrial superoxide levels by MitoSOX™ Red fluorescence.

(I) Flow cytometric analysis of intracellular ROS levels using DCFH‑DA.

(J) Quantification of ferroptosis‑related biochemical markers.

Data are presented as mean ± SD from n=3 independent biological replicates. Staining was quantified across at least 5 random fields per group using ImageJ. Two-tailed unpaired Student’s t-test or one-way ANOVA followed by Tukey’s post hoc test was used (**p*<0.05, ***p*<0.01, ****p*<0.001).

Supplementary Figure 10. Molecular and histological evidence for the synergistic downregulation of SLC40A1, suppression of pathological angiogenesis, and inhibition of fibrosis by the combinatorial therapy in scar tissues.

(A) Immunofluorescence staining and quantitative analysis of SLC40A1 protein in tissue sections.

(B) Immunohistochemical staining for the microvascular marker CD31 and the activation marker α-SMA.

(C) mRNA expression levels of fibrosis-related genes (TGFB1, COL1A1, COL3A1, ACTA2).

(D) Schematic of experimental groups.

(E) Western blot analysis of SLC40A1 and NEURL1 protein expression.

(F) Histopathological analysis. Representative H&E staining (upper panel) and Masson's trichrome staining (lower panel) of scar tissues.

(G) Measurement of oxidative stress and ferroptosis-related markers.

(H) mRNA expression levels of fibrotic markers.

(I) Immunohistochemical (IHC) analysis. IHC staining for CD31 (angiogenesis marker) and α-SMA (myofibroblast marker).

Complex means MNs@Exo@siSLC40A1@Cur

Data represent mean ± SD (n=6 mice per group). For histological, IF, and IHC quantifications in (A, B, F, I), at least 5 randomly selected non-overlapping visual fields (200× or 400×) per tissue section were quantified blindly using ImageJ/Fiji. Comparisons were evaluated by one-way ANOVA followed by Tukey’s post hoc test (**p*<0.05, ***p*<0.01, ****p*<0.001).

Supplementary Figure 11. The microneedle-based therapy exhibits favorable in vivo safety profiles without inducing significant organ damage or hepatic/renal dysfunction.

(A) Representative Hematoxylin and Eosin (H&E)-stained sections of major organs (heart, liver, spleen, lung, kidney) from mice in each treatment group, and the body weight change curve.

(B) Serum biochemical parameters (ALT, AST, UREA, CREA) of the mice.

(C) Expression levels of the pro‑inflammatory cytokines IL‑1β, IL‑6, and TNF‑αmRNA determined by qRT‑PCR.

(D) Cell viability assessed by CCK‑8 assay.

Complex means MNs@Exo@siSLC40A1@Cur

Data are presented as mean ± SD (n=6 mice per group for *in vivo* safety assays in A–C; n=3 independent biological repeats for *in vitro* cytotoxicity in D). Statistical analyses were performed using one-way ANOVA followed by Tukey’s post hoc test (ns = not significant, *p* > 0.05).
